# Perceived barriers to children’s active commuting to school: a systematic review of empirical, methodological and theoretical evidence

**DOI:** 10.1186/s12966-014-0140-x

**Published:** 2014-11-18

**Authors:** Wenhua Lu, E Lisako J McKyer, Chanam Lee, Patricia Goodson, Marcia G Ory, Suojin Wang

**Affiliations:** Silver School of Social Work, New York University, 20 Cooper Square, Room 240, 1 Washington Square, N, New York, NY 10003 USA; Department of Health & Kinesiology, Texas A&M University, College Station, TX 77843-4243 USA; Department of Landscape Architecture and Urban Planning, College of Architecture, Texas A&M University, College Station, TX 77843-3137 USA; Department of Health & Kinesiology, Texas A&M University, College Station, TX 77843-4243 USA; Health Promotion & Community Health Sciences, Texas A&M Health Science Center, School of Public Health, 1266 TAMU, College Station, TX 77843-1266 USA; Department of Statistics, Texas A&M University, 3143 TAMU, College Station, TX 77843-3143 USA

**Keywords:** Active commuting to school, Perceived barriers, Methodological quality, Theory utilization

## Abstract

Active commuting to school (ACS) may increase children’s daily physical activity and help them maintain a healthy weight. Previous studies have identified various perceived barriers related to children’s ACS. However, it is not clear whether and how these studies were methodologically sound and theoretically grounded. The purpose of this review was to critically assess the current literature on perceived barriers to children’s ACS and provide recommendations for future studies. Empirically based literature on perceived barriers to ACS was systematically searched from six databases. A methodological quality scale (MQS) and a theory utilization quality scale (TQS) were created based on previously established instruments and tailored for the current review. Among the 39 studies that met the inclusion criteria, 19 (48.7%) reported statistically significant perceived barriers to child’s ACS. The methodological and theory utilization qualities of reviewed studies varied, with MQS scores ranging between 7 and 20 (Mean =12.95, SD =2.95) and TQS scores from 1 to 7 (Mean =3.62, SD =1.74). A detailed appraisal of the literature suggests several empirical, methodological, and theoretical recommendations for future studies on perceived barriers to ACS. *Empirically*, increasing the diversity of study regions and samples should be a high priority, particularly in Asian and European countries, and among rural residents; more prospective and interventions studies are needed to determine the causal mechanism liking the perceived factors and ACS; future researchers should include policy-related barriers into their inquiries. *Methodologically*, the conceptualization of ACS should be standardized or at least well rationalized in future studies to ensure the comparability of results; researchers’ awareness need to be increased for improving the methodological rigor of studies, especially in regard to appropriate statistical analysis techniques, control variable estimation, multicollinearity testing, and reliability and validity reporting. *Theoretically*, future researchers need to first ground their investigations in theoretical foundations; efforts should be devoted to make sure theories are used thoroughly and correctly; important theoretical constructs, in particular, need to be conceptualized and operationalized appropriately to ensure accurate measurement. By reviewing what has been achieved, this review offered insights for more sophisticated ACS studies in the future.

## Introduction

Childhood obesity has become a global epidemic, with its increasing prevalence in both developed and developing countries [1–3]. Active commuting to school (ACS), defined as the use of active means such as walking or biking to and from school, may increase children’s daily physical activity and help them maintain a healthy weight [4–6]. Despite the significant health implications of ACS, the rates of ACS have declined over the past few decades [7]. In the United States (U.S.), for example, the percentage of children who walked or biked to school declined from 47.7% in 1969 to 12.7% in 2009 [7]. Similarly, in Australia, the percentage of children aged 5–9 who walked to school decreased from 57.7% in 1971 to 25.5% in 2003 [8].

To reverse the declining trend of ACS, one of the first crucial steps is to identify barriers that prevented children from walking or biking. Research in this area has expanded in the past 10 years, and studies have identified various perceived barriers related to children’s ACS [9–11]. Nevertheless, it is not clear whether and how these studies are methodologically sound and theoretically grounded. A rigorous assessment of existing literature is important because studies with poor designs, methodological flaws, or theoretical weaknesses could result in biased results and consequently render the subsequent interventions less effective.

In ACS research, perceived barriers can be defined as a person’s estimated level of challenges related to personal, environmental, social, and policy obstacles to ACS [12]. As a social cognitive construct, perceived barriers have been widely used or incorporated in health behavior theories, including the Health Belief Model, Social Cognitive Theory, Theory of Planned Behavior, and Social Ecological Theory [13–16]. Previous research has suggested that, compared with objective factors, e.g., urban form, individuals’ perceptions of the environment around them have a stronger and more direct relationship with children’s active commuting behavior [17]. Given the theoretical and empirical importance of perceived barriers in ACS research, it is essential to ensure that this construct is considered properly.

Therefore, the purpose of this systematic literature review was to critically assess the current literature on perceived barriers to children’s ACS. Specifically, we aimed to 1) examine research on perceived barriers to ACS, 2) identify different types and measures of perceived barriers reported by researchers, 3) assess the methodological quality of empirical studies on perceived barriers to ACS, and 4) evaluate the level of theory utilization in the studies, i.e., to what extent theory was used and how the construct of perceived barriers was conceptualized and operationalized. Empirical, methodological and theoretical recommendations for future studies will also be provided.

## Methods

### Search strategy

Following the PRISMA guidelines [18], we systematically searched for peer-reviewed articles related to perceived barriers to children’s ACS in the following six databases: Academic Search Complete, Eric, Medline, EMBASE, CINAHL Plus with Full Text, and SportDis. We chose these databases because they are comprehensive and include multidisciplinary journals. Different combinations of the following search terms were used: child, school child, adolescent, teen, or youth; elementary school, middle school, junior school, intermediate school, or high school; commute, travel, journey, walk, bike, cycle, bicycle, skateboard, or transport; to school. Specific terms used in the search were obtained from reviews of literature and the librarians’ and researchers’ expertise, and the search was adapted to match the specific structure of each database. A supplemental search was also conducted by reviewing the reference lists of the identified articles to further identify any relevant articles missed in the key word searches. Internal and external duplicates among the databases were examined and excluded in the process of article retrieval. In this review, *children* refers generally to children, adolescents, and young people aged 4 to 19, and *active commuting to school (ACS)* is a generic term for both active commuting/transport to *and* from school.

### Inclusion and exclusion criteria

To be eligible for inclusion in the review, the articles had to a) be published in a peer-reviewed English journal; b) include children (4- to 19-year-olds) and/or related adults (e.g., parent, teacher) as participants; c) be about active commuting, e.g., walking, biking, skateboarding, not passive commuting; d) have school as the origin or destination of active commuting; e) present empirical studies; f) use ACS as the outcome variable; and g) investigate perceived barriers to ACS, rather than objective barriers only. Further, we focused only on studies that used quantitative measures to examine perceived barriers for the present review to facilitate the process of synthesizing and comparing. A separate systematic review is in progress to analyze the findings of the qualitative studies. The date of the last search was February, 2013, and we limited the search to all studies published before that date.

### Data extraction

Data from the reviewed articles were abstracted using Garrard’s matrix method of literature review in health science [19]. Information extracted from each article included study characteristics (e.g., study area/setting, study design), participant characteristics (e.g., sample size, children’s age/grades), research methods (e.g., independent/dependent variables, data collection/analysis methods), and main findings (e.g., rates of ACS, identified perceived barriers to ACS). To ensure the credibility of data extraction, the first author and another researcher (both with research methods training) drew a sample of 16 articles (41%) and extracted the data independently. The researchers agreed on approximately 90% of the extracted data, indicating high inter-rater reliability.

### Methodological quality assessment

The authors tailored a methodological quality scale (MQS) for the current review based on previously established instruments [20–26] and the characteristics of the reviewed studies. For example, school characteristics was not included in any previous instruments, but it is an important consideration in ACS research: Findings from studies conducted in multiple locations are more generalizable, compared with those obtained from single location studies. All studies were assessed on 11 methodological criteria listed in Table 1. Possible points ranged from 4 to 24 with a higher number indicating greater methodological rigor. Each study’s point was first rated by the first author and then reviewed by another researcher majored in Statistics and trained in research methodology. Disagreements were resolved by discussion until agreement was reached.

**Table 1:** **Criteria for assessing studies’ methodological quality**

**Methodological criterion**	**Description**	**Score**
Study design	Experimental study (e.g., randomized control trial)	4
	Case control study	3
	Longitudinal study	2
	Cross-sectional study	1
Sample size	Large (>300)	3
	Medium (>100 and <300)	2
	Small (<100)	1
Definition of ACS	Defined	1
	Not defined	0
Data analysis	More advanced statistics (e.g., mixed models)	4
	Regression/analysis of covariance	3
	Bivariate statistics (e.g., ANOVA, Pearson *r*, *t* test)	2
	Descriptive only (e.g., frequency)	1
Control variable(s)	Included	1
	Not included	0
Multicollinearity testing	Tested	1
	Not tested/not mentioned	0
Data reliability testing	Reported results, based on other & own data (including reported elsewhere)	3
	Reported results, based on own data (including reported elsewhere)	2
	Reported results, based on other data	1
	Not reported	0
Data validity testing	Reported results, based on other & own data	3
	Reported results, based on own data	2
	Reported results, based on other data	1
	Not reported	0
Participant recruitment	Parent and child pair	2
	Parent, child or others (e.g., principals)	1
Participant characteristics	Reported (e.g., child age or grade)	1
	Not reported	0
School characteristics	Reported (e.g., size or composition), multiple locations	2
	Reported, single location	1
	Not reported	0

### Theory utilization assessment

A theory utilization quality scale (TQS) was created based on previously developed instruments [27,28] and tailored for the current review. For example, previous instruments assessed the conceptualization of perceived barriers by two scales: 1 = Reported, mentioned, or described; 0 = No report, mention, or description [27,28]. In this review, we added another scale in between (i.e., contextually described, but within a broader category), considering that perceived barriers were embedded, rather than clearly stated, in broader perceived environmental and social characteristics in some ACS studies. The reviewed studies were evaluated following the criteria described in Table 2. We first assessed whether and to what extent the authors used theories in the studies. For example, studies that proposed a conceptual framework based on previous theories and clearly measured related constructs received the highest score. In contrast, studies that did not clearly identify a theory but inferred, or studies that claimed to use a theoretical framework to guide the overall study design but did not provide evidence for it received a lower score. By using this scoring scheme, we focused mainly on the description of how theory was used in a study, rather than assessing whether or not a particular theoretical framework was considered as appropriate for investigating perceived barriers to ACS. We also evaluated how the construct of perceived barriers was conceptualized and operationalized in the reviewed studies. According to the criteria described in Table 2, we gave a higher score to studies that provided a clear definition of perceived barriers or described contextually what they meant by perceived barriers in the case of ACS. In contrast, studies that did not define the term clearly received a lower score. Similarly, studies that reported how they operationalized perceived barriers and clearly described the measured items were scored higher, while studies that claimed they measured perceived barriers but did not describe the measured items were scored lower. The possible range of the theory utilization assessment scores was 0 to 7. To examine the reliability of code and the assessment by the first author, two additional researchers trained in health behavior theories scored a sample of 10 articles (26%) independently. The sample of articles was selected randomly by using Microsoft Excel’s random sorting function. The raters agreed on 93% and 90% with the original code, respectively, indicating good inter-rater reliability. Discrepancies found were addressed by re-appraisals and discussions, or judgment by a fourth party, until consensus was reached.

**Table 2:** **Criteria for assessing studies’ theory utilization**

**Criterion**	**Description**	**Examples**	**Score**
**Did the authors use theory in their studies?**			
Theory utilization	Clear identification/ operationalization of theory/constructs used	A conceptual framework was proposed based on a theory and measured constructs/variables accordingly.	3
	Inferred theory or partial use of theory	A theory was not clearly identified, but three or more theoretical constructs of a theory were measured.	2
		A theory was identified but only one or two constructs of the theory were measured.	
	May be informed by theory/slight evidence of use of theory	The use of a theoretical framework was claimed to guide design, program, or measures, but was not evidenced.	1
		A theory was not clearly identified, but one or two theoretical constructs of a theory were measured.	
	No evidence of using theory		0
**What did the authors mean by “perceived barriers” in each article?**			
Conceptualization of perceived barriers	Defined or contextually described	A clear definition of “perceived barriers” was provided.	2
		What “perceived barriers” mean in the case of active commuting to school was clearly described.	
	Contextually described, but within a broader category	Participants’ perceived environmental characteristics that may influence children’s ACS were described, which included both perceived facilitators and barriers.	1
	Not defined/described		0
**Did the authors describe/detail how “perceived barriers” were measured?**			
Operationalization of perceived barriers	Clearly operationalized /reported	Different items were used to measure “perceived barriers” and the items were clearly described.	2
	Somewhat/slightly operationalized	Different items were claimed to be used to measure “perceived barriers”; however, the items were not described.	1
		“Perceived barriers” were claimed to be measured; however, it’s not clear what items were used.	
	Not reported/described		0

After the assessments were finished for both methodological quality and theory utilization quality, a correlation test between the MQS and TQS scores obtained were conducted to establish the relationship between them. This study was considered exempt by the institutional review board at Texas A&M University.

## Results

**Figure 1:**
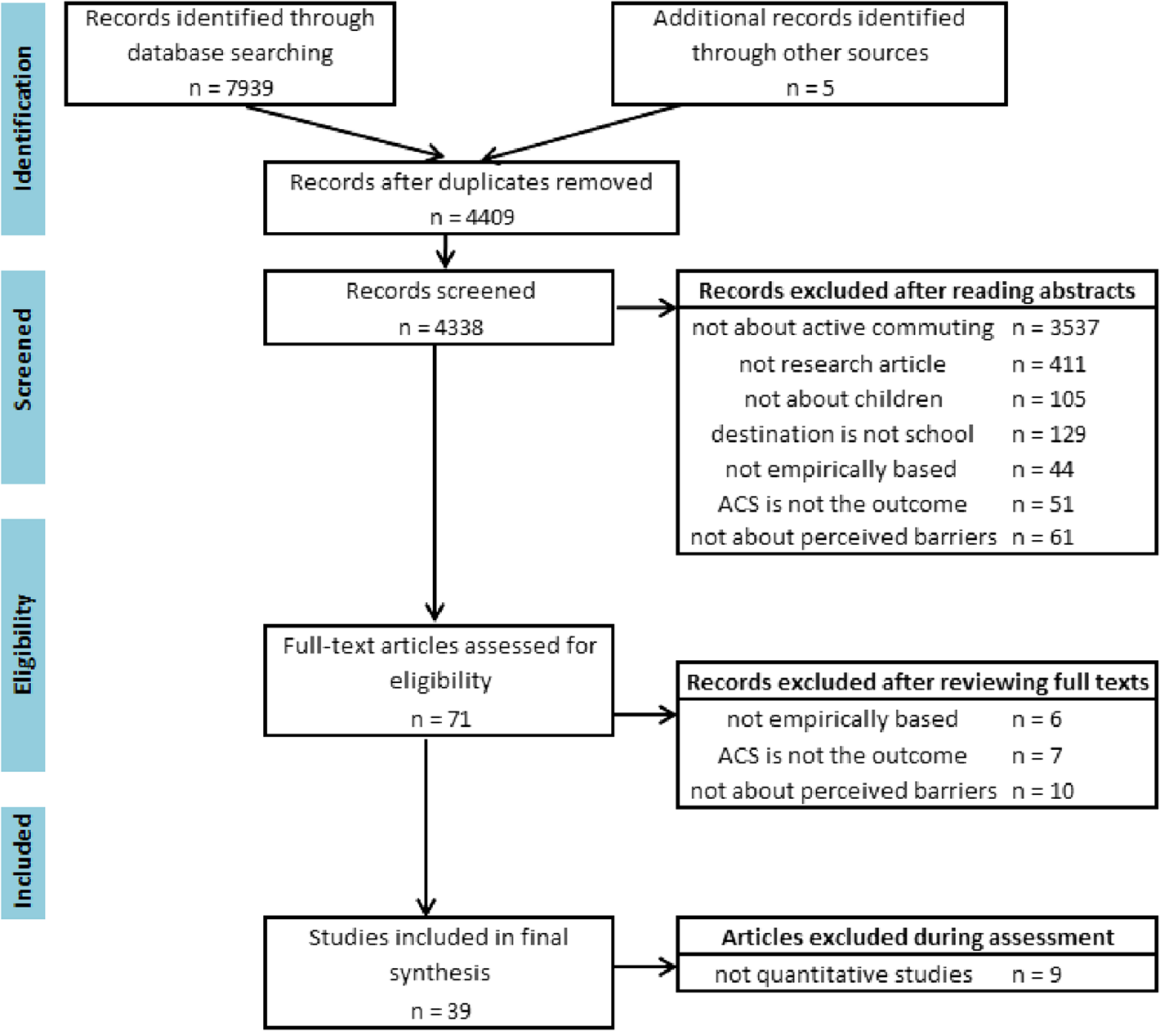
**Search and selection of articles.**

A total of 4,409 unique records were identified from six databases and additional manual searching (Figure 1). More than 4,300 articles were excluded after the abstract review, of which the majority were not about ACS (n =3,537). After examining the full text of 71 articles, 23 were eliminated because they were not empirically based, did not use ACS as the outcome, or were not about perceived barriers. Nine of the remaining articles were further excluded as they were purely qualitative investigations. The final analysis consisted of 39 articles [5,28–66] that met all inclusion criteria (Table 3).

**Table 3:** **Characteristics of studies on perceived barriers of children’s active commuting to school (N = 39)**

**Lead author/year/country**	**Journal**	**Sample size**	**Children's grades/Ages/ethnicity**	**Independent variables/Program**	**Select findings**
**Babey (2009), US**	*Journal of Public Health Policy*	3,893 parent–child pairs	12-17 years	Individual, family, and environmental characteristics with ACS	(1) Rate of ACS: 49.8% walked, biked or skateboarded to or from school at least once a week, 25% ACS 3 or more days per week.
					(2) Correlates of ACS: distance (−), male (+), Latino (+), from lower-income families (+), attending public school (+), and living in urban areas (+); parental supervision (−), and parent knowing little or nothing about adolescents’ whereabouts after school (+).
**Bringolf-Isler (2007), Switzerland**	*Preventive Medicine*	1,345	1st, 4th, 8th graders	Personal and family factors, environmental data (GIS)	(1) Rate of ACS: 77.8%.
					(2) Predictors for non-active commuting: child’s age (+), number of cars in the household (+), daycare attendance (+), parental safety concerns (+), and belonging to French-speaking population (+).
**Carson (2010), Canada**	*Revue Canadienne De Sante Publique*	3421 parent–child pairs	5th grade	Socio-demographic characteristics, parental perceptions of neighborhood environment.	(1) Rate of ACS: 39%.
					(2) Predictors of ACS: neighborhood with high perceived sidewalks/parks (+).
**Carver (2005), Australia**	*American Journal of Health Promotion*	345 parent–child pairs	12-13 years	Socio-demographic characteristics, parental perceptions of neighborhood environment.	(1) Rate of ACS: Walking for boys: 39%; walking for girls: 46%; biking for boys: 10% (17/172); biking for girls: 1% (2/175).
					(2) Predictors of ACS: For boys: no significant bivariate associations between perceptions of the neighborhood and boys’ walking to/from school; For girls: having friends living in the neighborhood (+), lots of other boys/girls to “hang out” with (+) and parents’ concerns about busy traffic (−).
**D’Haese (2011), Belgium**	*International Journal of Behavioral Nutrition and Physical Activity*	696	6th grade	Distance, criterion distance (i.e., cumulative percentages of children commuting to school by bike, on foot, and in a passive way, per covered distance), and environmental perceptions	(1) Rate of ACS: 38.1% by bike, 21.1% walk.
					(2) Correlates of ACS: Perceived accessibility to walk (+).
**Emond (2011), US**	*Journal of Transport Geography*	1,357	10th-12th graders	Socio-demographics and attitudinal factors (individual factors, social-environment factors, and physical-environment factors), distance (home location geo-coded)	(1) Rate of biking: 32.7% to school, 33.4% from school.
					(2) Correlates of biking: perceived bicycling comfort (+), parental encouragement (+), perceived distance (−), having to cross a freeway (−), confidence in one’s bicycling ability (+), being males (+).
**Evenson (2006), US**	*International Journal of Behavioral Nutrition and Physical Activity*	480	6th and 8th graders	Socio-demographics, perceived safety, aesthetics, and facilities near the home; parental provision of transportation.	(1) The 24 individual items on safety, aesthetics, facilities near the home, and transportation mostly indicated fair to moderate reliability. (2) Predictors of ACS: Perceived neighborhood safety concern (“walkers and bikers on the streets in my neighborhood can easily be seen by people in their homes”) (−); more physical activity facilities (+).
**Fries (2012), US**	*Advances in Transportation Studies an international journal*	12,613	Kindergarten through 8th grade	N/A	(1) Rate of ACS: 14.8%.
					(2) Top parental perceived barriers for urban and suburban children: intersection safety and traffic speed/volume. Distance from school affected suburban students more than urban students.
**Fulton (2005), US**	*Research Quarterly for Exercise and Sport*	1,395 parent–child pairs	4th grad through 12th grade	Demographics, body mass index, behavioral, psychosocial, attitudinal, and environmental characteristics.	(1) Rate of ACS: 14%.
					(2) Predictors of ACS: having sidewalks (+), boys (+), lower grades (+).
**Heelan (2008), US**	*Journal of Physical Education, Recreation & Dance*	150	School age	Seven categories of perceived barriers to ACS.	(1) Predictors of ACS: whether or not the child wanted to actively commute (+), having enough time (+), busy streets (−), child maturity (+), carpool availability (−), and crosswalks (−).
					(2) Perceived barriers of ACS by frequency: safety concerns, busy streets, weather, time, convenience.
**Hume (2007), Australia**	*American Journal of Health Promotion*	280	10 year olds, grade 5	Perceived physical and social environmental characteristics	(1)Frequencies of walking to/from school per week for boys: 2.07, for girls: 1.66.
					(2) Perceived barriers of ACS for boys: number of accessible destinations in the neighborhood (+).
					(3) Perceived predictors of ACS for girls: having a neighborhood that was easy to walk/cycle around (+) and perceiving lots of graffiti (+).
**Hume (2009), Australia**	*American Journal of Preventive Medicine*	309	Children aged 5–6 and children aged 10-12	Demographics, individual-level predictors, social environmental predictors, physical environmental predictors	(1) Rates of ACS: Walking 2.9 mean trips/week, biking 0.4 mean trips/week; ACS 1–5 trips/week: 39.7%; ACS daily 22.3%.
					(2) ACS significantly increased between 2004 and 2006 among children and adolescents.
					(3) Predictors of ACS: children of parents who reported that the child had many friends in their areas (+), adolescents whose parents perceived insufficient traffic lights and pedestrian crossings in their neighborhood (−), adolescents of parents who were satisfied with the number of pedestrian crossings (+).
**Kerr (2006), US**	*Medicine & Science in Sports & Exercise*	259	5-18 years old	Objective measures, including the neighborhood and individual walkability index, and subjective measures, including socio-demographic variables and perception of the local environment (e.g., residential density, street connectivity, and crime safety).	(1) Rate of ACS: 18.1% walked or biked 5 days a week, and 25.1% actively commuted at least once a week.
					(2) Correlates of ACS: Parent concerns and neighborhood aesthetics were independently associated with ACS. Perceived access to local stores and biking or walking facilities accounted for some of the effect of walkability on ACS.
**Lee (2013), US**	*Annals of Behavioral Medicine*	601 parent–child pairs	Hispanic predominant	Environmental perceptions about walkability, safety concerns, and parental attitudes and preferences	(1) Parental attitudes and children’s preferences were associated with the odds of walking.
					(2) Safety concerns (traffic danger, stranger danger, and getting lost) were higher among drivers, but only significant in bivariate analyses.
**Loucaides (2010), Cyprus**	*Central European Journal of Public Health*	1966	Grades 1-12	Personal, social and environmental characteristics	(1) Rates of ACS: 19.4%.
					(2) Predictors of ACS: having enough time in the morning to walk to school (+) and parents feeling that it was safe for children to walk to school (+), and long distance from home to school (−).
**McMillan (2007), US**	*Transportation Research Part A*	1128	Grades 3-5	Urban form demographics, caregivers’ beliefs, perceptions and attitudes about travel by different modes, household demographics	Correlates of ACS: urban form (+), perceived neighborhood safety concerns (−), perceived traffic safety concerns (−), household transportation options (+), caregiver valuing social interaction (+), caregiver reporting driving more convenient (−), social/cultural norms (+), and socio-demographics (−).
**Mendoza (2010), US**	*Journal of Applied Research on Children: Informing Policy for Children at Risk*	149	Grade 4, Latino subsample	Socio-demographics, child self-efficacy, parent self-efficacy, parent outcome expectations, perceived neighborhood safety, observed pedestrian safety behaviors	(1) Rate of ACS: 43%.
					(2) Predictors of ACS: parent self-efficacy (+) for the full sample, parent outcome expectations (+) for Latino children.
					(3) ACS was positively associated with daily moderate-to-vigorous physical activity.
**Mendoza (2011), US**	*Pediatrics*	149	Grade 4	Socio-demographics, child self-efficacy, parent self-efficacy, parent outcome expectations, perceived neighborhood safety, observed pedestrian safety behaviors	(1)Acculturation (+) and parent outcome expectations (+) were significantly associated with the change in percent active commuting.
					(2) Positive associations between active commuting and physical activity.
**Merom (2006), Australia**	*Health& Place*	812	5-12 years	Socio-demographics, parents’ perceptions about safe environment, child’s enjoyment of walking, and perceived health benefits of ACS, child’s level of independence, parents’ modes of transport to work	(1) Rate of frequent ACS: 37%; Rates of regular ACS: 22%.
					(2) Predictors of ACS: distance (−), child’s age (+), parental perceptions of road safety (−), and attending public school (+).
**Miller (2013), US**	*American Journal of Health Behavior*	74 parent–child pairs	Grades 1-6	Age, designated time periods, gender, parent vs. child, normal weight vs. overweight	(1) Children were most active after and least active before and during school.
					(2) Weight was not related to activity.
					(3) Boys were more confident than girls, whereas parents felt more confident than children did about active transport.
**Mota (2007), Portugal**	*Annals of Human Biology*	705	Grades 7-12	Socio-economic position, environmental assessment, including connectivity of the street network, infrastructure for walking and cycling, neighborhood safety, and social environment.	(1) Rate of ACS: 52.6%.
					(2) Predictors of ACS: occupational status of mother (−) and father (−), father’s educational level (−), street connectivity (+), father’s occupation (+), perceived presence of four-way intersections (+).
**Nelson (2010), Ireland**	*Journal of Physical Activity and Health*	2159	15 to 17 years	Socio-demographics, perceived physical environmental characteristics	(1) Rates of ACS: 61.3% walked and 8.7% cycled.
					(2) Correlates of ACS in the final model for boys: perceived land-use-mix diversity (+), perceived presence of public parks (+); for girls: traffic safety (−), visibility (+), the presence of cycle tracks (+), and the ease of walking/cycling to transit (+).
**Panter (2010), England**	*Journal of Epidemiology and Community Health*	2012	9-10 years	Socio-demographics, attitudes, perceptions, and social support.	(1) Rates of ACS: 54%; 40% walking and 9% biking.
					(2) Correlates of ACS: boy (+) for biking, girl (+) for walking, distance less than 1 km (+), mothers ACS (+), parental attitude (+), parental safety concerns (−), the presence of social support from parents and friends, (+), parental perceived neighborhood walkability (+).
**Price (2011), US**	*Journal of School Health*	314	N/A	respondents type, school type, respondents’ perceptions of ACS factors	(1) Top 3 factors of ACS by frequency: distance to school, traffic speeds, and traffic volume.
					(2) Several participants expressed concerns about liability issues related to students’ ACS.
					(3) Some reported that schools are not responsible for students’ safety once students leave school grounds.
**Ridgewell (2009), Australia**	*Urban Policy and Research*	248 students, 128 parents	8-11 years	N/A	Rates of ACS: 21.0% walking to school, 25.3% walking from school; 4.7% biking to school, 4.3% biking from school.
**Rodriguez (2009), US**	*Journal of School Health*	1,897	Grades 3-5	Socio-demographics, environmental factors, access factors, attitude factors	(1) Rates of ACS: 11.1% walked, 1.4% biked.
					(2) Predictors of ACS: age (+), perceptions that walking saves time (+), distance (−), car ownership (−), access to a school bus (−).
**Rojas-Guyler (2007), US**	*Californian Journal of Health Promotion*	71	N/A	Principals’ beliefs conducive to children and health.	(1) Rate of ACS: Mean percentage of ACS was 11.77%.
					(2) The number of students using ACS did not significantly differ between schools with a restrictive policy and schools with no restrictive policy. Principals at schools with higher ACS rates were significantly more likely to report that students should consider ACS if residing within one mile, had significantly more enabling environments, and had significantly less restrictive environments.
**Rossen (2011), US**	*Journal of Physical Activity and Health*	365	Grades 3-5	Street block-residence characteristics, individual-level characteristics, perceived safe neighborhood etc.,	(1) Rate of ACS: 56% walked.
					(2) Predictors of ACS: distance to school (−) and level of incivilities (+). (3) High levels of neighborhood incivilities were associated with lower levels of perceived safety.
**Salmon (2007), Australia**	*American Journal of Health Promotion*	720	4-13 years	Socio-demographics	(1) Rate of ACS: 41%.
					(2) Predictors of ACS: individual (“child prefer to be driven” (−), “no time in the mornings” (−); social (“worry child will take risks” (−), “no other children to walk with” (−), “no adults to walk with” (−), and environmental barriers (“too far to walk” (−), “no direct route” (−). Positive association: “concern child may be injured in a road accident” and ACS (+).
**Schlossberg (2006), US**	*Journal of the American Planning Association*	292	Grades 6-8	Distance from school on the street network, five measures of perceived urban form: intersection density, dead-end density, route directness, major roads, and railroads, and measures of perceived convenience (e.g., desire to drop a child off on the way to work, backpack is too heavy)	(1) Rates of ACS: 15% to school, 25% from school.
					(2) Predictors of ACS: distance (−), intersection density (−), dead ends (−).
					(3) Reported perceived barriers by frequency: ease of dropping child off on the way to work, the heaviness of the child’s backpack, bad weather, dangerous traffic conditions, high-speed vehicles, lack of complete sidewalks.
**Silva (2011), Brazil**	*Journal of Physical Activity and Health*	1672	11 to 17 years	Socio-demographics, type of school attended, time spent, and perceived barriers.	(1) Rate of ACS: 62.7%.
					(2) Predictors of frequent use of ACS: long distance (−), and traffic (−).
					(3) Predictors of modes of transport: long distance (−), crime (−), and traffic (−).
**Timperio (2006), Australia**	*American Journal of Preventive Medicine*	912 (235 families of children aged 5 to 6; 677 families of children aged 10 to 12)	Two groups: 5 to 6 years; 10 to 12 years	Personal factors, family factors, SES, parent-perceived social/physical neighborhood, child-perceived social/physical neighborhood, objective measures of route to school	(1) Rates of ACS: 47.8% walked for children aged 5–6, 60.4% walked for those aged 10–12; 6.6% biked for children aged 5–6 and 6.3% for those aged 10–12; Either walked or biked: 48.9% for children aged 5–6 and 62.0% for those aged 10–12.
					(2) No gender difference among younger children; boys cycled more than girls in older children.
					(3) Correlates of ACS: parental perception of few other children around (−) and no lights or crossings (−), and objectively assessed busy road barrier en route to school (−). For younger group, objectively assessed variables (−); older group: good connectivity (−). For both group, route 800 meters (+).
**Trapp (2011), Australia**	*International Journal of Behavioral Nutrition and Physical Activity*	1197 parent–child pairs	Grades 5-7	Individual, social, perceived environmental, objective environmental factors.	(1) Rates of ACS: 31.2% for boys, and 14.6% for girls.
					(2) Predictors of ACS: school neighborhood design (in boys) (+), parental confidence in their child’s cycling ability (+), parental perceived convenience of driving (+), parental perceptions regarding neighborhood safety issues (i.e., whether the neighborhood is safe enough and the need to cross busy roads ) (−) and child’s preference to cycle (for both boys and girls) (+).
**Van Dyck (2010), Belgium**	*International Journal of Behavioral Nutrition and Physical Activity*	1,281	17.1 ± 0.5 years	Socio-demographics, physical environmental perceptions, psychosocial factors	(1) Rates of ACS: 6.6% walked, 51.8% cycled.
					(2) Predictors of ACS: gender (−), smoking status (−), higher walkability of the neighborhood (+) and more social modeling (+).
**Yeung (2008), Australia**	*Transportation Research Part A*	318	8 vs. 10 years	Anthropometric characteristics (self-reported), distance (self-reported), and perceived barriers, including safety issues and physical infrastructure.	(1) Rate of ACS: 1/3.
					(2) Predictors of ACS: commuting distance (−).
**Zhou (2010), US**	*Journal of Transportation Safety & Security*	347 students, 2551 parents	75% elementary (Kindergarten-5th grade)	Demographics, and subjective variables (e.g., school attitudes, enjoyment, and health)	(1) Rates of ACS: 8.9% (child reported), 9.5% (parent reported).
					(2). Students living in different distance intervals are subject to different barriers.
					(3) Security and safety remain the primary factors of concern for parents to allow their children to ACS, esp. for those living at short walkable distances
					(4) School, parents’ and students’ attitudes, grade levels, and allowable grade level all had significant impact on the students’ walking/biking rates.
**Zhu (2008), US**	*Child Health and Human Development*	1281	Grades 1-5	Personal factors, social factors, and parents’ perception of the physical environment	(1) Walking was a typical mode for 28% and 34% of trips to and from school, respectively, and mostly accompanied by an adult.
					(2) Correlates of ACS: parental education level (−), car ownership (−), child and parental personal barriers (−), and school bus availability (−), and positive peer influences (+); environmental factors, including proximity to school (+), safety concerns (−) and the presence of highway or freeway en route (−).
**Zhu (2009), US**	*Journal of Public Health Policy*	2695	Grades 1-5	Personal, social, and physical environmental factors.	(1) Walking was a typical mode for 27.8% and 31.5% for the trips to and from school, respectively.
					(2) Correlates of ACS: Personal and social factors, including parental education (−), car ownership (−), personal barriers (−), and school bus availability (−), parental and child positive attitude and regular walking behavior (+), and supportive peer influences (+); Environmental factors, including distance (−), safety concerns (−), presence of highways/freeways(−), convenience stores (−), office buildings (−), and bus stops en route(−).
**Ziviani (2004), Australia**	*Occupational Therapy International*	164	Grades 1-7	Socio-demographics, psychosocial factors, perceived environmental factors, children's level and enjoyment of physical activity, and perceived importance of physical activity	(1) Mean number of days walking to school in a week was 1.00 ± 1.62.
					(2) Predictors of ACS: perceived importance of physical activity, parents’ individual history of transport to school, distance, concern about traffic, and concerns about personal safety.

Note: *ACS* = Active Commuting to School; (+) means positive correlation with outcome measures; (−) means negative correlation with outcome measures.

### Characteristics of reviewed studies

Table 3 outlines the select information extracted from the 39 reviewed articles. These articles represented 30 peer-reviewed journals from varying disciplines, including health (n =33, 84.6%), transportation (n =4, 10.3%), and urban planning (n =2, 5.1%). Most articles (n =24, 61.5%) were written by researchers from health-related fields, with seven articles (17.9%) representing collaborative work of researchers across disciplines (e.g., public health and urban planning). All identified articles were published after 2004, with the numbers increasing almost annually.

The studies were undertaken in 10 countries, i.e., the U.S. (n =20, 51.3%), Australia (n =10, 23.1%), Belgium (n =2, 5.1%), Canada (n =1, 2.6%), Switzerland (n =1), Cyprus (n =1), Portugal (n =1), Ireland (n =1), England (n =1), and Brazil (n =1). Regarding study settings, 15 (38.5%) were conducted in urban areas, 4 (10.3%) included participants from both rural and urban areas, one (2.6%) was undertaken in rural area, and the remaining studies (n =19, 48.7%) did not specify study settings or distinguish between urban or rural areas. Sample sizes of the reviewed studies varied from 74 to 12,613, and most studies were exploratory (n =36, 92.3%) rather than hypothesis-driven (n =3, 7.7%).

### Active commuting to school

The definitions of ACS were not consistent across the studies. Most studies defined ACS as walking or biking to school *usually* (n =32, 82.1%), while some defined it as walking or biking to school at least once a week (n =3, 7.7%). Other definitions of ACS included walking or biking to school *ever*, walking or biking to school the longest portion of the journey to school, and walking or biking to school 5 days a week. Similarly, the dependent variable, i.e., ACS, was measured differently across the studies. Most studies used a dichotomized dependent variable as active versus non-active (n =24, 61.5%), or the frequency of ACS as a continuous variable (n =10, 25.6%). Eight studies (20.5%) did not report the rates of ACS. For studies that measured walking, biking, and other modes of transports, such as skateboarding, together as the usual mode to/from school (n =19, 48.7%), the rates of ACS ranged between 11.8% [54] and 77.8% [30]. For studies that considered/reported walking or biking separately as the usual mode to/from school (n =12, 30.8%), the rates of walking were from 6.6% [61] to 61.3% [49] and the rates of biking were between 1% [32] and 51.8% [61]. Only two studies focused specifically on biking to school [34,60].

### Perceived barriers to ACS

Fourteen studies (35.9%) did not find any statistically significant (significant for short hereafter) perceived barriers to child’s ACS in their analyses. For the other 25 studies, we further excluded four studies (10.3%) that reported perceived barriers based on descriptive or bivariate statistics [36,51,52,63], one study that measured single item (i.e., perceived safety) [47], and one study that used summary index (i.e., 11 items for parental concerns with the mean calculated) [41].

Among the remaining 19 studies (48.7%) that reported significant results, six studies included personal barriers, including parents’ lack of time, ease of dropping child off the way to work, child’s heavy backpack, child’s preference to be driven to school, and walking as requiring too much planning ahead; 18 studies reported perceived physical environmental barriers, among which traffic safety and distance were most commonly cited; and 10 studies identified different types of perceived social environmental barriers to ACS, which were centered on neighborhood safety (Table 4).

**Table 4:** Summary of statistically significant* perceived barriers identified in reviewed studies (n = 19)

Personal barriers (n = 6)**	**Physical environment barriers (n = 18)**	**Social environment barriers (n = 10)**
No time [56,65]	Traffic safety (e.g., speed, volume) [32,35,44,49,50,57,58,66]	Neighborhood safety [42,44,60]
Ease of dropping child off the way to work [42,57]	Distance [34,43,56,58,66]	Stranger danger [64]
Heaviness of the child’s backpack [57,64]	Freeway/highway/crosswalks [34,38,42,64]	Crime/danger [58]
Child’s preference of being driven to school [56]	Road safety [30,46]	Graffiti [39]
Walking as requiring too much planning ahead [42]	Bad weather [57,64]	Worry child will take risk [56]
	Busy street [38]	No other child to walk with [56]
	No direct route [56]	No adults to walk with [56]
	Lack of sidewalks [57]	Few children around [59]
	No/insufficient lights or crossings [40,59]	Getting lost [64]
		Stray dogs [64]
		Exhaust fume [64]
		Personal safety [66]
		Concern about something happening to child on the way [50]

Note: *p < .05. **Number of studies that identified the categories of perceived barriers.

Eleven of the 19 studies that identified significant predictors of ACS used/included children’s surveys, and, unanimously, traffic safety was regarded as a barrier to ACS among children. Compared with children, parents were more concerned about neighborhood safety, e.g., crime, strangers, and stray dogs. In regard to children’s characteristics, 12 of the 19 studies focused on elementary/primary school children, five sampled middle/high school adolescents only [34,35,49,57,58], and two recruited both elementary and middle school students [29,43]. For middle school students, the identified perceived barriers were mostly about physical environmental characteristics, including distance, traffic safety, bad weather, and lack of sidewalks; no personal barriers were reported for middle school students. In contrast, perceived barriers for elementary school children were more diverse, including various personal, social environmental, and physical environmental characteristics.

### Methodological quality of reviewed studies

The methodological quality of reviewed studies varied, with the MQS scores ranging between 7 and 20 (Mean =12.95, SD =2.95) (Table 5). Most studies employed a cross-sectional study design and used a survey instrument to collect the data (n =36, 92.3%). For data analysis, 26 (66.7%) utilized regression or analysis of covariance; seven employed more advanced statistics (17.9%), e.g., mixed models; and six used bivariate or descriptive statistics (15.4%). Over half of the studies (n =22, 56.4%) included control variables in the data analysis, and the most commonly included control variables were distance, participants’ sociodemographics such as race/ethnicity, gender, and educational level, and school site. Moreover, 27 studies (69.4%) tested multicollinearity among the variables, and 12 studies (30.8%) did not mention any testing performed for the multicollenarity issue.

**Table 5:** **Distribution of methodological quality characteristics across reviewed studies (N = 39)**

**Methodological Criterion**	**Description**	**Score**	***n*** **of studies**	**Percentage (%)**
Study design	Experimental study (e.g., Randomized control trial)	4	1	2.6
	Case control study	3	1	2.6
	Longitudinal study	2	1	2.6
	Cross-sectional study	1	36	92.3
Sample size	Large (>300)	3	29	74.4
	Medium (>100 and <300)	2	8	20.5
	Small (<100)	1	2	2.6
Definition of ACS	Defined	1	38	97.4
	Not defined	0	1	2.6
Data analysis	More advanced statistics (e.g., mixed models)	4	7	17.9
	Regression/analysis of covariance	3	26	66.7
	Bivariate statistics (e.g., ANOVA, Pearson *r*, *t* test)	2	3	7.7
	Descriptive only (e.g., frequency)	1	3	7.7
Control variable(s)	Included	1	22	56.4
	Not included	0	17	43.6
Multicollinearity testing	Tested	1	27	69.2
	Not tested/not mentioned	0	12	30.8
Data reliability testing	Reported results, based on other & own data (including reported elsewhere)	3	9	23.1
	Reported results, based on own data (including reported elsewhere)	2	9	23.1
	Reported results, based on other data	1	6	15.4
	Not reported	0	15	38.5
Data validity testing	Reported metrics, based on other & own data	3	0	0.0
	Reported metrics, based on own data	2	4	10.3
	Reported, based on other data	1	6	15.4
	Not reported	0	29	74.4
Participant recruitment	Parent and child pair	2	12	30.8
	Parent, child or others (e.g., principals)	1	27	69.2
Participant characteristics	Reported (e.g., child age or grade)	1	37	94.9
	Not reported	0	2	5.1
School characteristics	Reported (e.g., size or composition), multiple locations	2	26	66.7
	Reported, single location	1	2	5.1
	Not reported	0	11	28.2

Many studies (n =15, 38.5%) did not report on the method or result of the data reliability assessment. Nine studies (23.1%) reported data reliability based on another study’s data and their own data, including those reported elsewhere. Nine studies (23.1%) reported the reliability based solely on their own data, and another six studies reported (15.4%) the metrics based on other studies’ data. Among the studies that reported reliability metrics, eight (20.5%) conducted both internal consistency test and test-retest reliability test; seven (17.9%) performed internal consistency tests only; and six (15.4%) conducted test-retest reliability test only.

Likewise, most studies did not report the data validity testing (n =29, 74.4%). Only four studies (10.3%) reported validity testing based on their own data and six studies (15.4%) reported results from other studies. Among the studies that reported validity, four (10.3%) tested face validity, and four (10.3%) tested construct validity.

Regarding participants recruitment, 12 (30.8%) studies recruited parent/child pairs, and 27 (69.2%) recruited only children, parents, or other stakeholders. Two studies (5.1%) did not report any participant characteristics, and 11 studies (28.2%) did not present any information about the school characteristics. Among the studies that reported school characteristics, 26 had the participating schools at different locations, and two studies focused on a single school.

### Theory utilization of reviewed studies

The theory utilization scores of the reviewed studies ranged from 1 to 7 (Mean =3.62, SD =1.74). As shown in Table 6, 17 (43.6%) of the reviewed studies did not propose or test any theoretical model or show any evidence of theoretical uses. Sixteen studies (41.0%) clearly identified a theoretical model and measured part or all of the relevant constructs; four studies (10.3%) either inferred a theory or presented partial use of a theory; and two studies (5.1%) only showed some but often weak evidence of theory uses.

**Table 6:** **Distribution of theory utilization characteristics across reviewed studies (N = 39)**

**Criterion**	**Description**	**Score**	***n*** **of studies**	**Percentage (%)**
Theory utilization	Clear identification/operationalization of theory/constructs used	3	16	41.0
	Inferred theory or partial use of theory	2	4	10.3
	May be informed by theory/slight evidence of use of theory	1	2	5.1
	No evidence of using theory	0	17	43.6
Conceptualization of perceived barriers	Defined or contextually described	2	1	2.6
	Contextually described, but within a broader category	1	12	30.8
	Not defined/described	0	26	66.7
Operationalization of perceived barriers	Clearly operationalized	2	32	82.1
	Somewhat/slightly operationalized	1	5	12.8
	Not reported or described	0	2	5.1

Among the 16 studies that clearly identified a theoretical framework, 14 studies used the Social Ecological Model; one used the Theory of Reasoned Action; and one developed a modified theoretical model based on Social Ecological Theory and Social Cognitive Theory [44].

As to the conceptualization of perceived barriers, most studies (n =26, 66.7%) did not provide a definition of perceived barriers. Only one study (2.6%) provided a clear definition of perceived barriers and 12 studies (30.8%) described perceived barriers but within a broader category, e.g., perceived environmental characteristics which included both perceived facilitators and barriers. In contrast, most studies clearly described how they operationalized perceived barriers (n =32, 82.1%); five studies (12.8%) slightly operationalized the construct, e.g., not indicating what items were used to measure perceived barriers; and two studies (5.1%) did not include any description on the operationalization method (Table 6).

The correlation between MQS and TQS was statistically significant (r = .581, p < .001), indicating a positive relationship between the methodological quality and quality of theory utilization of the reviewed studies.

## Discussion

The aim of this systematic literature review was to summarize and critically assess the current literature on perceived barriers to children’s ACS. To our knowledge, this is the first systematic review evaluating methodological quality and theory utilization of empirical studies on perceptions of children’s ACS. A detailed appraisal of the literature suggests several empirical, methodological, and theoretical issues.

### Empirical issues

The results of our analysis revealed a need for more ACS studies globally. Most of the studies identified were conducted in the U.S. or Australia. There is a need for more studies to better understand the roles of perceived barriers to ACS in other areas, e.g., Asia and Europe. Although the international literature showed higher rates of ACS in several Asian countries, e.g., the Philippines and China, shifts to more passive commuting modes were anticipated in these countries with continued modernization and increasing car ownership [67,68]. Given that childhood obesity has become a global epidemic, promotion efforts for ACS should begin immediately in Asian countries. Although our findings indicated that compared with U.S. or Australia, the rates of ACS were generally higher in European countries (e.g., Switzerland, Portugal, Ireland, and England), most of the European studies recruited small samples from one area, which limited the representativeness of their findings. Therefore, more evidence from large-scale empirical data on ACS within a European country is warranted, as well as studies conducted in more European countries. Considering the diverse environmental characteristics of European cities/countries and that individuals’ health behavior can be influenced by characteristics of the geographical area where they live [69], there might be wide variations in perceived barriers to ACS across European countries/cities. With limited studies conducted in areas other than the U.S. and Australia, such comparisons are not meaningful, if not impossible. Future studies using well-established instruments tailored for specific populations are needed in regions other than those reported in this review.

Also, there is a need for future research to consider/report walking or biking separately as the usual mode to/from school; around 70% of the reviewed studies examined perceived barriers to the two commuting modes together. To exacerbate the problem, most of the other 30% of studies that examined the two modes separately concentrated on walking, with little attention given to biking. Walking and biking are different behaviors, and, therefore, perceived predictors of biking are very likely to differ from those of walking [60]. More empirical knowledge about perceived barriers specifically to biking to school is required.

This review also highlighted a shortage of ACS studies regarding perceived barriers in rural settings. Among the 39 studies identified, only five studies clearly stated the inclusion of rural locations. The roles of environmental or social characteristics on ACS may vary across different community settings. In terms of rural/urban designation, distinctive natural and living environments of these two areas may illuminate different perceived barriers to ACS, and serve as ideal contexts for natural experiments to make such comparisons. However, few comparative studies examined such potential variations. Given that rural residents are less likely to meet physical activity recommendations compared with urban or suburban residents [70], more work is needed on ACS that specifically focuses on rural–urban variations. It is also worth noting that the criteria for rural–urban classification are different across countries. In England, for example, areas are defined as rural if they fall outside of settlements with more than 10,000 resident population, and six rural categories are classified, including town and fringe, village, and hamlet and isolated dwellings [71]. In comparison, urban areas must encompass at least 50,000 people in the U.S., and rural areas encompass all population, housing, and territory not included within an urban area [72]. Understandably, the inconsistent definitions of urban and rural areas across countries may result in a mismatch, i.e. resulting in a methodological issue influencing the association between perceived barriers and ACS, and consequently affect the accurate comparison between the two areas.

Third, more prospective and intervention studies with perceived barriers as predictors of ACS changes are needed. Most of the reviewed studies were cross-sectional, which cannot infer cause-and-effect relationships. To influence policy changes and large-scale environmental interventions, evidence from intervention studies is crucial [73]. Further, prospective studies conducted at a minimum of three time points are recommended, because studies with two observation points are limited in drawing firm conclusions on the direction of the relationships among study variables [74]. It is possible that participants’ perceptions of the environment might be influenced by the increased level of ACS at the second point, e.g., after an intervention was conducted [75].

In regard to perceived barriers identified by previous studies, our findings underscored the lack of inquiries into participants’ perceptions on policy/regulatory barriers. Most research on participants’ perceived barriers to ACS used a couple of established instruments that focused on factors at the personal, physical and social environment levels, thus leaving policy as an under-researched area. Policy issues can influence individuals’ decision-making regarding ACS. For example, different countries or districts may have different school siting or school choice policies, which can influence their commuting distance and availability of viable travel modes [76,77]. Individual schools may also have opposing school bus policies that discourage ACS, e.g., grade/age minimums for ACS or policies requiring parents to designate their child as a walker or a rider [77,78]. Identification of participants’ perceived policy barriers could inform possible policy changes in support of ACS, while neglect of these potential barriers may result in less effective interventions.

### Methodological issues

Assessment of the methodological quality of the reviewed studies raised several methodological and analytical concerns. One major limitation was the lack of consistent definition for ACS. Great variation was observed in the proposed definitions and measurement of ACS. Although many studies defined ACS as walking or biking to school *usually*, researchers did not clarify what “usually” means, e.g., whether it’s over 3 days a week or 4 days a week. Some studies defined ACS as walking or biking at least *once* a week. Moreover, when used as the dependent variable, ACS was measured categorically in some studies but continuously in others, e.g., as frequency of ACS or percentage of ACS children, which compromised the generalizability of identified perceived correlates. Although there’s no “golden rule” for defining ACS, researchers should at least provide a valid rationale for the use of specific definitions and measurements of ACS. For example, health researchers who are more interested in the relationship between ACS and health outcome may prefer more detailed or rigorous measurements such as frequency and duration of ACS, which are more relevant for long-term health benefits [10]. In this case, dichotomizing ACS may be less appropriate.

Second, multiple studies applied univariate or bivariate statistical techniques and failed to justify their applications. When these techniques are used to analyze the association between multiple determinants and an outcome variable, biased or misleading results may be produced. To correctly assess the complicated relationships among the variables, we need more sophisticated methods which allow for modeling multiple variables and diverse pathways among them. Further, given that most ACS data are school-based or district-based, we recommend researchers resort to multilevel or hierarchical techniques that can effectively separate individual-level effects from cluster-level effects [79]. Advanced statistical techniques may not be necessary for all research questions, but researchers need to provide valid rationale for using simpler methods in multivariate cases. Otherwise, results should be interpreted with caution.

Also, most studies that conducted correlation tests did not include or report the inclusion of control variables in their analyses. Leaving out important control variables can cause model specification bias and render the interpretation of results suspicious [80]. Lack of a theoretical basis may account for the lack of control variable(s) in data analysis, as the selection of control variables is mainly theory-driven. Although control variables can also be chosen based on the statistical tests, we recommend ACS researchers to utilize theory to more effectively conceptualize the multi-level constructs related to behavioral outcomes. For those who included control variables, socioeconomic factors and distance were the most common variables. Researchers may also be interested in how the association between perceived barriers and ACS is modified by other objective environmental characteristics such as neighborhood walkability and land use types. Previous research has demonstrated the relative influence of some urban form variables on the probability of a child walking or biking to school [44]. Although individuals’ perceptions of the environment around them have been suggested as a stronger predictor of children’s active commuting behavior than physical environment [17], including and testing objective measures as mediators can further strengthen existing evidence and provide empirical support for more cost-effective interventions. To achieve this goal, collaborations among scholars from various disciplines such as public health, urban planning, and transportation are encouraged.

Another concern was the lack of reporting multicollinearity diagnostics in the studies. In the presence of multicollinearity, regression estimates are unstable. Multicollinearity can misleadingly inflate the standard errors of coefficients and make some variables statistically insignificant when they should be significant otherwise [81]. Moreover, when multicollinearity exists, the simultaneous analysis of interrelated constructs may yield spurious or confounded results whereby it is impossible to distinguish the individual effects. To minimize the risk of multicollinearity, researchers should avoid including predictors that are conceptually identical, regardless of the sample size. Other alternatives dealing with multicollinearity include ridge regression, combining of independent variables into a single index, or conducting factor analysis [81,82]. It is also possible that some researchers tested multicollinearity but didn’t report the diagnostics in their papers. To confirm the audience of the studies’ methodological rigor, we suggest that researchers report multicollinearity testing in their papers.

The quality of the reviewed studies was further compromised by the authors’ neglect of reliability and validity testing. Most studies either did not mention data reliability/validity or reported the test result based on previous studies’ data. Reliability and validity testing is critical because measurement errors can directly affect the results and their interpretation [83]. Researchers can either evaluate the score reliability and validity using their own samples or rely on published sources [84]. However, reliability and validity evidence from established instruments is applicable only if researchers use the same instrument in the same form and the instrument has been validated in a population similar to their samples [85]. Published reliability/validity coefficients may not be generalizable to a particular sample under consideration [84]. Despite the importance of reporting reliability and validity testing, many journals do not include specific requirements for empirical studies to report psychometric properties of the instrument being used and scores being analyzed. To facilitate the publication of high quality research, we recommend that journals refine their editorial guidelines and require authors to report reliability and validity coefficients for the data being analyzed. Researchers’ awareness regarding the roles of reliability and validity also need to be increased to ensure the correct interpretation of their results.

### Theoretical issues

The level of theory utilization among the reviewed studies was low. Over half of the studies were not theoretically driven or used theories superficially. Theories provide a framework for identifying determinants of particular health behaviors, which constitutes a critical initial step in the development of successful interventions [86]. The lack of theoretical basis might account for the overarching number of exploratory studies among the reviewed studies, which typically assume only their direct effects on ACS without considering interaction among predictor variables. The lack of theory use posed an added concern regarding “kitchen sink” regressions in which any variables available were included. When selecting a variable, its theoretical relevance should be as important as, if not more important than, its statistical significance. The relatively low level of theory utilization suggests that health behavior studies need to advance further in sophistication of study designs [28]. To overcome this shortcoming, researchers need to raise their awareness of using theories, not only in funding application but also for manuscript development. Journals may also need to expand the word limits they placed on manuscript submissions to ensure researchers have enough space to elaborate on theory utilization [27,28,87]. Despite the importance of theory use, it was possible that some researchers did not use any theory because the research area was rather new with no earlier model as a basis; therefore, they considered their studies as exploratory, rather than hypothesis-driven.

Our findings also highlighted the common use of the Social Ecological Models (SEM). All except two of the reviewed studies that identified a theoretical framework used SEM. Our result was in line with findings from previous reviews of physical activity research that SEM has been the most commonly adopted theoretical framework [88,89]. SEM provides a comprehensive framework for understanding the multi-level determinants of health behaviors [15,90]. Recently, researchers have used SEM to support a new emphasis on environmental causes of behaviors [86,89]. While the consistent use of the SEM facilitated the process of synthesizing and comparing findings, the SEM lacks sufficient specificity regarding specific characteristics at each level. Consequently, other significant factors that may work with hypothesized factors at each level may be neglected. For example, perceived barriers as a personal level construct may be influenced by other social cognitive factors at the same level such as attitudes, self-efficacy, and intention; neglecting these constructs may result in an incomplete picture and consequently biased results. Unfortunately, these important social cognitive constructs were rarely investigated within the ACS context [11]; it might be time to put these factors back into equation.

Another weakness of the research was the divergence between conceptualization and operationalization of perceived barriers. Only one study clearly defined perceived barriers; most authors simply assumed that readers knew what “perceived barriers” meant. With this assumption, most of the studies skipped the conceptualization stage and directly operationalized perceived barriers by describing survey items that were used to measure the construct. When a construct is poorly conceptualized, it is very unlikely that the construct is properly operationalized. To make the situation even worse, most of the reviewed studies did not conduct a validity test. Consequently, the quality of construct measurement and the interpretation of results were questionable. For future ACS studies, improving the conceptualization and operationalization of investigated constructs should be a high priority.

### Implications for practice

The statistically significant association between MQS and TQS of the reviewed studies not only confirmed the internal consistency of the instruments that we developed, but also had great implications for future research on perceived barriers to ACS. When researchers used theory to guide inquiry, they tended to utilize more sophisticated analytical techniques. Similarly, when researchers resorted to more advanced statistical methods in their data analyses, they were more likely to ground their research questions in theory. If the reciprocal relationship between theory use and data analysis holds true, then the low level of theory use and generally undesirable methodological quality of the reviewed studies raised an important practical question as to the reliability of their findings: Can policy makers trust the perceived barriers identified by the researchers and design ACS interventions accordingly? It appears the research in this field still holds room for improvement, and its quality could be considerably improved if researchers (1) pay more attention to the theoretical grounding of their inquiry, and (2) improve the methodological rigor of studies.

### Limitations and strengths

This review is not without limitations. First, we limited our search to articles published in English, and therefore relevant literature published in other languages was excluded. Second, with the heterogeneity in the definition of ACS and the absence of standardized measurement tools of perceived barriers, inter-study comparisons must be considered with caution. Third, we chose to focus on perceived barriers in this review mostly because perceived barriers is an important construct in many health theories and represents one of the most commonly investigated constructs in ACS research. Future reviews are warranted to assess how and to what extent other theoretical constructs or measures were considered in literature. For example, perceived facilitators is also an important construct in ACS research and does not necessarily mean the opposite of perceived barriers. Also, objective measures, though not well represented in commonly used theories, were widely investigated in ACS studies, and the methodological and theoretical issues we identified in this review might influence their effect on ACS as well. Furthermore, this review was limited by the relatively small sample of studies to evaluate trends in theory use over years and to compare studies by sub-groups or disciplines. Despite the limitations, the strengths of this review need to be recognized. First, it used an extensive search strategy to locate articles in six databases and rigorously screened articles through well-defined inclusion/exclusion criteria. Second, the instruments that we developed for assessing the methodological and theoretical qualities of existing ACS literature were based on well-established instruments and tailored for ACS studies. The instruments served well to capture existing discrepancies in literature and provided detailed insight for future studies.

## Conclusions

Following rigorous assessment process, this systematic review has provided a detailed discussion of empirical, methodological, and theoretical issues in the current literature of active transport, in regard to perceptions of barriers preventing children from ACS. Based on our findings and in light of the limitations of this review, we have several empirical, methodological, and theoretical recommendations for advancing the quality of future studies on perceived barriers to ACS.

*Empirically*, increasing the diversity of study regions and samples should be a high priority, particularly in Asian and European countries, and among rural residents. More studies are also needed to examine walking and biking as separate active commuting behaviors. Regarding the relation between individual perceptions and ACS behavior, more prospective and interventions studies conducted at multiple time points are needed to determine the causal mechanism liking the perceived factors and ACS. Moreover, future researchers should also include policy-related barriers into their inquiries. *Methodologically*, the conceptualization of ACS should be standardized or at least well rationalized in future studies to ensure the comparability of results. Favorably, definitions of ACS need to reflect the frequency and magnitude of the behavior more accurately. Second, researchers’ awareness need to be increased for improving the methodological rigor of studies, especially in regard to appropriate statistical analysis techniques, control variable estimation, multicollinearity testing, and reliability and validity reporting. *Theoretically*, future researchers need to first ground their investigations in theoretical foundations. Further, efforts should be devoted to make sure theories are used thoroughly and correctly. Important theoretical constructs, in particular, also need to be conceptualized and operationalized appropriately to ensure accurate measurement. By reviewing what has been achieved, we hope this review offers insights for more sophisticated active transport studies in the future.
